# Lethal lung hypoplasia and vascular defects in mice with conditional *Foxf1* overexpression

**DOI:** 10.1242/bio.019208

**Published:** 2016-09-16

**Authors:** Avinash V. Dharmadhikari, Jenny J. Sun, Krzysztof Gogolewski, Brandi L. Carofino, Vladimir Ustiyan, Misty Hill, Tadeusz Majewski, Przemyslaw Szafranski, Monica J. Justice, Russell S. Ray, Mary E. Dickinson, Vladimir V. Kalinichenko, Anna Gambin, Paweł Stankiewicz

**Affiliations:** 1 Department of Molecular & Human Genetics, Baylor College of Medicine, Houston, TX 77030, USA; 2 Program in Translational Biology & Molecular Medicine, Baylor College of Medicine, Houston, TX 77030, USA; 3 Department of Neuroscience, Baylor College of Medicine, Houston, TX 77030, USA; 4 Institute of Informatics, University of Warsaw, Warsaw 02-097, Poland; 5 Division of Pulmonary Biology, Cincinnati Children's Hospital Research Foundation, Cincinnati, OH 45229, USA; 6 Department of Pathology, University of Texas MD Anderson Cancer Center, Houston, TX 77030, USA; 7 Genetics & Genome Biology Program, SickKids, Toronto, Ontario M5G 0A4, Canada; 8 Department of Molecular Physiology & Biophysics, Baylor College of Medicine, Houston, TX 77030, USA

**Keywords:** Lung development, Pulmonary vasculature, ACDMPV

## Abstract

*FOXF*1 heterozygous point mutations and genomic deletions have been reported in newborns with the neonatally lethal lung developmental disorder, alveolar capillary dysplasia with misalignment of pulmonary veins (ACDMPV). However, no gain-of-function mutations in *FOXF1* have been identified yet in any human disease conditions. To study the effects of *FOXF1* overexpression in lung development, we generated a *Foxf1* overexpression mouse model by knocking-in a Cre-inducible *Foxf1* allele into the ROSA26 (R26) locus*.* The mice were phenotyped using micro-computed tomography (micro-CT), head-out plethysmography, ChIP-seq and transcriptome analyses, immunohistochemistry, and lung histopathology. Thirty-five percent of heterozygous R26-Lox-Stop-Lox (LSL)-*Foxf1* embryonic day (E)15.5 embryos exhibit subcutaneous edema, hemorrhages and die perinatally when bred to *Tie2*-cre mice, which targets *Foxf1* overexpression to endothelial and hematopoietic cells. Histopathological and micro-CT evaluations revealed that R26*Foxf1; Tie2-cre* embryos have immature lungs with a diminished vascular network. Neonates exhibited respiratory deficits verified by detailed plethysmography studies. ChIP-seq and transcriptome analyses in E18.5 lungs identified *Sox11*, *Ghr*, *Ednrb*, and *Slit2* as potential downstream targets of FOXF1. Our study shows that overexpression of the highly dosage-sensitive *Foxf1* impairs lung development and causes vascular abnormalities. This has important clinical implications when considering potential gene therapy approaches to treat disorders of FOXF1 abnormal dosage, such as ACDMPV.

## Abstract (precis)

**Summary:** Similar to *Foxf1* loss, *Foxf1* overexpression in mice is lethal. This finding highlights the need to consider alternatives beyond gene therapy to find a cure for alveolar capillary dysplasia with misalignment of pulmonary veins (ACDMPV).

## INTRODUCTION

The *FOXF1* (Forkhead box F1) gene located on chromosome 16q24.1 encodes a member of the FOX family of transcription factors characterized by a distinct forkhead DNA binding domain, and plays an important role in epithelium-mesenchyme signaling as a downstream target of Sonic hedgehog (Mahlapuu et al., 2001; Dharmadhikari et al., 2015). *FOXF1* is expressed in fetal and adult lungs, placenta, and prostate (Hellqvist et al., 1996; Bozyk et al., 2011; van der Heul-Nieuwenhuijsen et al., 2009). In the mouse embryonic lungs, *Foxf1* expression is restricted to mesenchyme-derived cells such as alveolar endothelial cells and peribronchiolar smooth muscle cells (Kalinichenko et al., 2001a). Lung mesenchymal cells include numerous subtypes such as airway smooth muscle cells, fibroblasts, pericytes, vascular smooth muscle cells, and alveolar endothelial cells. Alveolar endothelial cells are differentiated vascular cells of mesenchymal origin, and line blood vessels that are in close proximity to air spaces in the lung.

Heterozygous point mutations and genomic deletions involving *FOXF1* or its upstream enhancer have been reported in newborns with a lethal lung developmental disorder alveolar capillary dysplasia with misalignment of pulmonary veins (ACDMPV, OMIM 265380) with or without defects involving heart, gastrointestinal, or genitourinary systems (Stankiewicz et al., 2009; Bishop et al., 2011; Sen et al., 2013). A majority of *Foxf1*^+/*−*^ mice die perinatally, exhibiting defects in lung vasculature, similar to those in patients with ACDMPV (Kalinichenko et al., 2001b; Stankiewicz et al., 2009).

*FOXF1* has also been reported to be epigenetically inactivated in breast and colorectal cancers (Lo et al., 2010; Mitchell et al., 2014) and overexpressed in Patched-associated tumors, including basal cell carcinoma, medulloblastoma, and rhabdomyosarcoma (Wendling et al., 2008a; Armeanu-Ebinger et al., 2011). *FOXF1* overexpression was found in lung fibroblasts from patients with idiopathic pulmonary fibrosis (Melboucy-Belkhir et al., 2014). Isolated gastrointestinal abnormalities such as pyloric stenosis, mesenterium commune, and aplasia of the appendix were found to be associated with 16q24.1 duplications involving *FOXF1*; however, RNA or protein lung studies could not be performed (Dharmadhikari et al., 2014). Hence, while effects of the loss of function of *FOXF1* in lung disease and cancer are well documented, effects of the overexpression of *FOXF1*, specifically in the context of lung development, are not currently known.

To study the consequences of *Foxf1* overexpression*,* we developed a Cre-inducible *Foxf1* allele by knocking-in *Foxf1* into the ROSA26 (R26) locus after a lox-STOP-lox (LSL) cassette (ROSA26-lox-STOP-lox–*Foxf1*). Here, we show that *Tie2*-cre-induced heterozygous R26*Foxf1* mice manifest a wide range of phenotypes that cause death from embryonic to perinatal stages due to lung and vascular defects. Determining dosage sensitivity of *Foxf1* is important to inform future gene therapy approaches to potentially treat patients with ACDMPV and other disease conditions due to genetic alterations in *FOXF1*.

## RESULTS

### *Tie2*-cre-mediated overexpression of *Foxf1* results in embryonic vascular and perinatal lung defects

We generated a floxed *Foxf1* construct to target the ROSA26 locus (R26-LSL-*Foxf1*; Fig. S1) as previously described (Hohenstein et al., 2008; Carofino and Justice, 2015). First, we tested overexpression of *Foxf1* in all tissues by crossing the R26-LSL-*Foxf1* and the *CMV*-cre lines. We found that it led to embryonic lethality of R26*Foxf1*; *CMV*-cre embryos, displaying hemorrhage at E12.5 (Fig. S2). *Foxf1* is endogenously expressed in mesenchyme-derived cell types such as endothelial and smooth muscle cells in the lungs (Kalinichenko et al., 2001a). To determine the effects of overexpression of *Foxf1* in a similar context, we mated R26-LSL-*Foxf1* mice to *Tie2*-cre mice, overexpressing *Foxf1* in the endothelial and hematopoietic lineages.

**Fig. 1.**
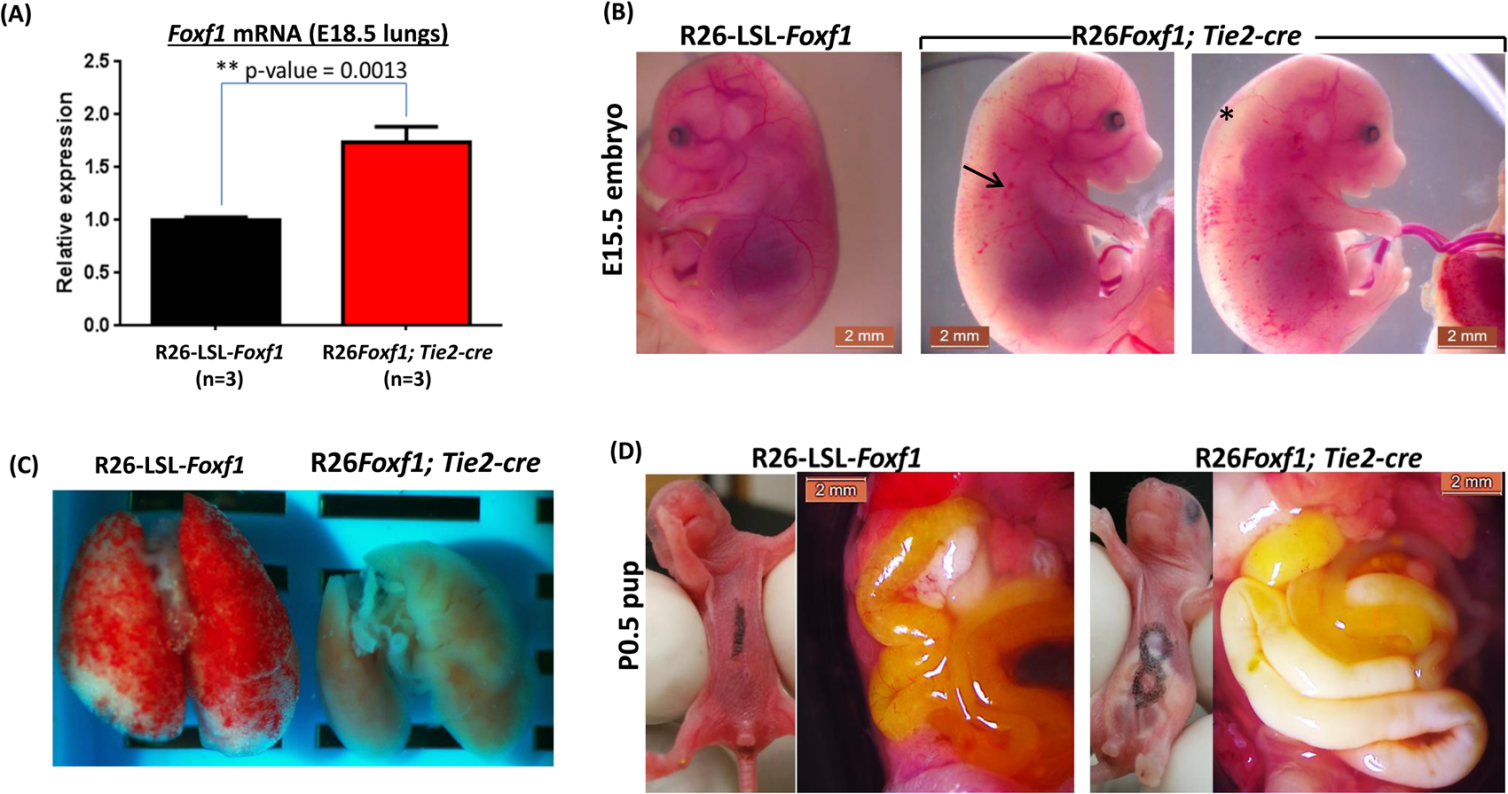
**Perinatal vascular and lung defects.** (A) *Foxf1* Taqman qRT-PCR shows ∼1.7-fold overexpression of *Foxf1* in the E18.5 lungs from the R26*Foxf1; Tie2-*cre embryos. (B) Normal E15.5 R26-LSL-*Foxf1* embryo and hemorrhages and edema seen in E15.5 R26*Foxf1; Tie2-*cre embryos; * indicates edema, and hemorrhages are indicated by an arrow. (C) Smaller lungs of a R26*Foxf1; Tie2*-cre P0.5 pup compared to the lungs from a R26*-LSL-Foxf1* control pup. (D) Normal abdominal and intestinal region in a control P0.5 R26-LSL-*Foxf1* pup compared to chylous abnormalities seen in the abdominal and intestinal region in P0.5 R26*Foxf1; Tie2-*cre pups.

Quantitative RT-PCR analysis showed that *Foxf1* was overexpressed 1.7-fold in R26*Foxf1; Tie2-*cre E18.5 lungs compared to R26-LSL-*Foxf1* control lungs (Fig. 1A). qRT-PCR on RNA isolated from flow-sorted pulmonary endothelial cells (*n*=2-3 lungs) similarly showed a trend towards overexpression of *Foxf1* compared to wild-type littermates (Fig. S3).

Data at E15.5, E18.5, and postnatal day (P)0.5 from timed matings showed a significant decrease in the number of heterozygous +/R26*Foxf1;+/Tie2*-cre (R26*Foxf1;Tie2*-cre) mice [i.e. from the expected 50% Mendelian frequency to ∼12% at P0.5, indicating embryonic lethality, however a majority survived to birth and died perinatally (Table 1)]. At E15.5, approximately 35% of R26*Foxf1*; *Tie2*-cre embryos exhibited edema and/or hemorrhages (Fig. 1B). Approximately 67% of R26*Foxf1;* *Tie2*-cre pups at P0.5 had smaller lungs compared to those of R26-LSL-*Foxf1* control littermate pups (Fig. 1C). Additionally, R26*Foxf1;* *Tie2*-cre pups that survived after birth displayed abdominal distention and chyle accumulation in the intestinal wall, suggesting lymphatic vascular defects (Fig. 1D), not found in R26-LSL-*Foxf1* littermate control pups. Complete blood counts (CBCs) at P1.5 revealed lower platelet counts in R26*Foxf1;* *Tie2*-cre pups compared to control pups (Fig. S4). Analysis of placental histology and determination of placenta weights at E18.5 showed no apparent placental defect (Fig. S5). Histological analysis of E18.5 liver sections revealed no differences between the two groups (data not shown). No lethality or adverse phenotypes were observed in *Tie2*-cre mice in the study. Additionally, crossing of the *Foxf1*^+/*−*^ mice with the *R26Foxf1;* *Tie2*-cre mouse line was not able to rescue the neonatal lethality observed in the *Foxf1*^+/*−*^ mice (data not shown). PCR analysis to detect Cre-mediated recombination is shown in Fig. S6.

**Table 1.**
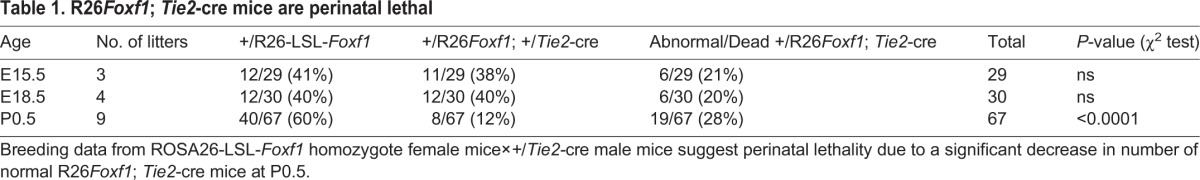
**R26*Foxf1*; *Tie2*-cre mice are perinatal lethal**

**Fig. 2.**
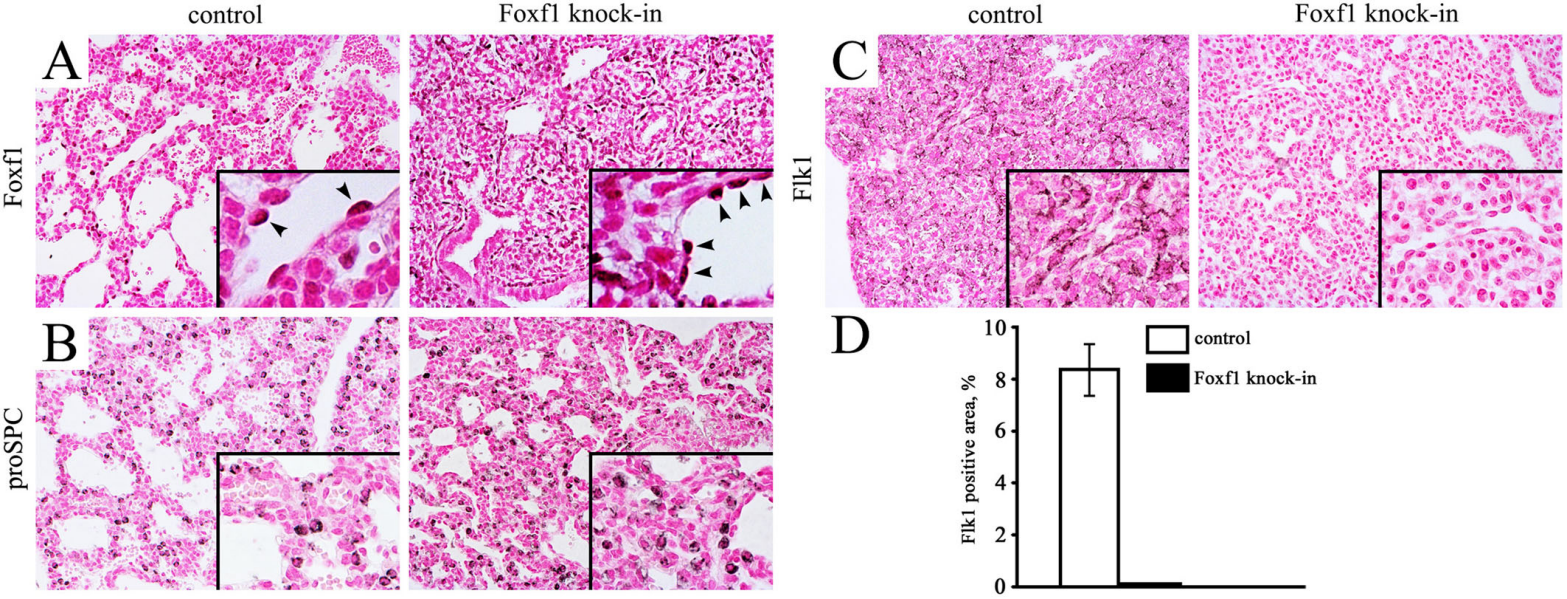
**Lung immaturity in R26*Foxf1; Tie2-*cre embryos.** (A) Immunohistochemistry shows diminished lung sacculation and increased FOXF1 staining in E18.5 R26*Foxf1; Tie2-*cre embryos compared to R26*-*LSL*-Foxf1* littermates (control). FOXF1-positive endothelial cells are depicted with arrowheads (inserts). Slides were counterstained with nuclear fast red. Images were taken at the same exposure. (B) ProSPC staining was unaltered whereas FLK1 staining was decreased in R26*Foxf1; Tie2-*cre lungs (C). Magnification is ×200 (inserts are ×400). (D) Quantification of FLK1 staining showing decreased FLK1 expression in R26*Foxf1*;*Tie2*-cre lungs. Data are shown as mean±s.e.m.

Histopathological evaluation showed immature lungs in E18.5 R26*Foxf*1:*Tie2*-cre embryos, and immunohistochemistry (IHC) analysis revealed increased FOXF1 staining in R26*Foxf*1: *Tie2*-cre lungs (Fig. 2A), a finding consistent with elevated *Foxf1* mRNA levels (Fig. 1A). Staining for epithelial-specific marker proSPC was unchanged (Fig. 2B). Lung immaturity was associated with a reduced capillary network as shown by diminished staining for FLK1, an endothelial marker (Fig. 2C,D). Immunostaining for smooth muscle marker α-SMA showed no major differences (data not shown).

### Lungs of R26*Foxf1*: *Tie2*-cre pups are hypoplastic

**Fig. 3.**
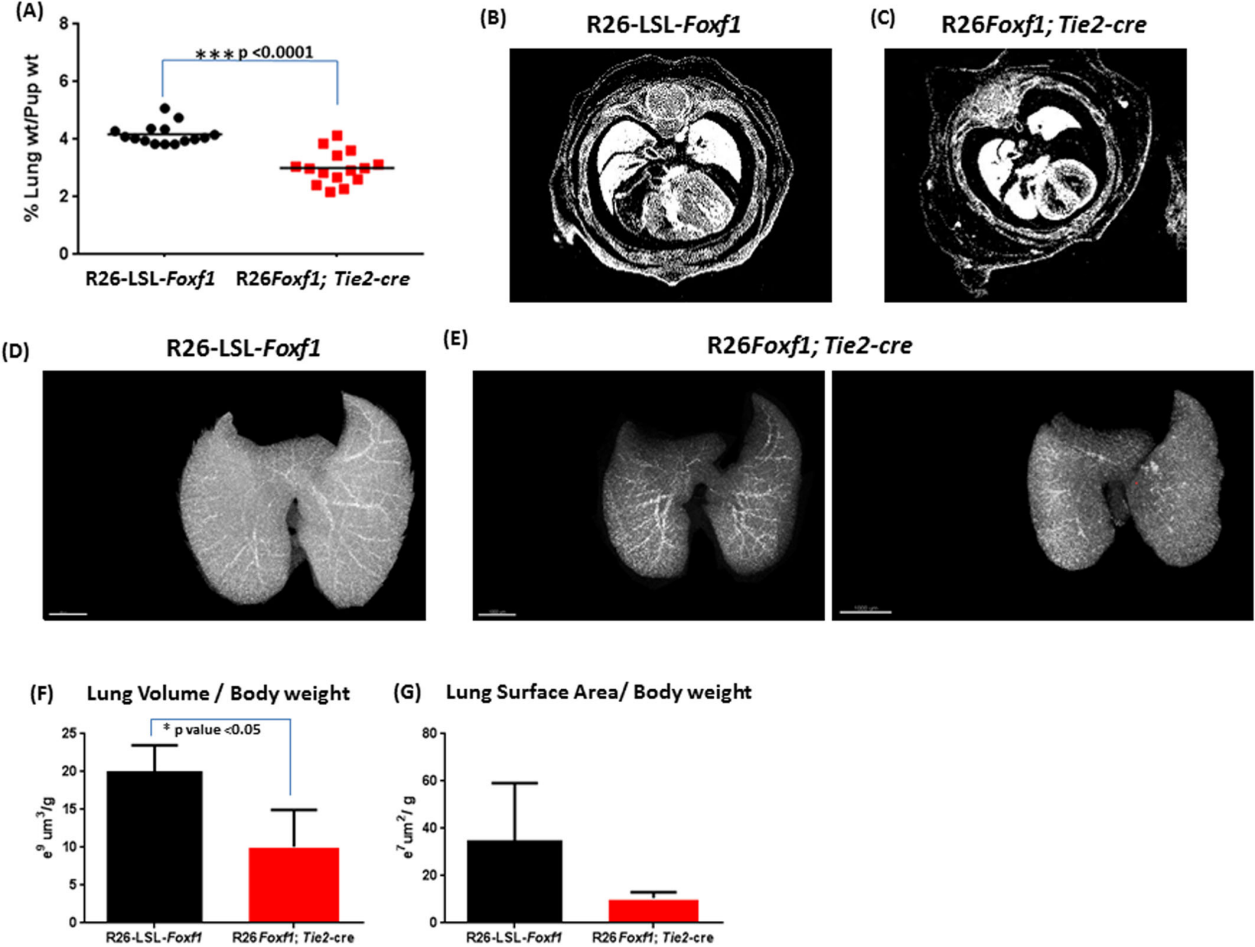
**Lung hypoplasia.** (A) Lower wet lung weight:pup weight ratios in the R26*Foxf1; Tie2*-cre P0.5 pups compared to R26*-LSL-Foxf1* control littermate pups. Horizontal line indicates mean wet lung weight:pup weight ratio. (B,C) Transverse sections comparing E15.5 R26*Foxf1; Tie2-*cre lungs to time-matched R26-LSL-*Foxf1* control lungs. (D,E) Representative E18.5 lung micro-CT images obtained after ROI analysis. Scale bars: 400 µm. (F,G) Quantification of micro-CT imaging data of E18.5 lung volumes and surface areas by surface rendering using Imaris (*n*=3 for both groups; data are shown as mean±s.e.m.).

At P0.5, the wet lung weight (LW) to body weight (BW) ratios of R26*Foxf1; Tie2-*cre pups were significantly lower (approximately 3.0%) compared to that of R26*-LSL-Foxf1* control littermate pups (approximately 4.1%) (Fig. 3A). Computational analyses of micro-CT imaging of E15.5 and E18.5 R26*Foxf1; Tie2-*cre embryos confirmed that their lungs are hypoplastic compared to R26-LSL-*Foxf1* control embryos (Fig. 3B-E), and did not show mispatterning of major pulmonary vessels (Fig. S7). Furthermore, the LV:BW ratios were significantly lower (approx. 50%) in E18.5 R26*Foxf1; Tie2-*cre embryos compared to controls (Fig. 3F). There was also a trend towards a lower ratio of lung surface area to body weight (approx. 30% lower) (Fig. 3G). Other organs, including the heart and liver, did not show any hematomas or evidence of blood pooling.

### R26*Foxf*1: *Tie2*-cre pups exhibit respiratory defects

**Fig. 4.**
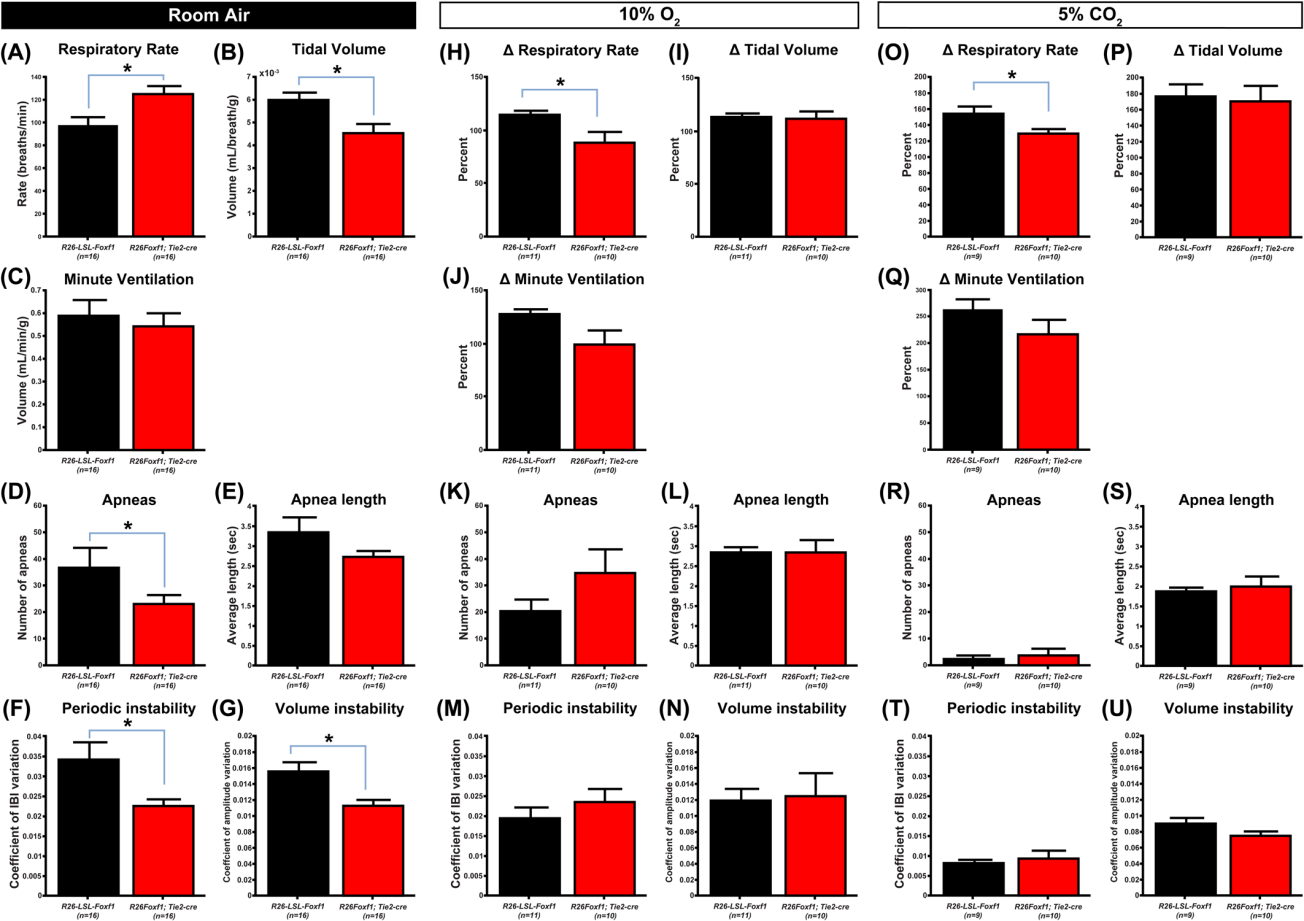
**R26*Foxf1; Tie2-*cre P0.5 neonates show respiratory deficits under room air, hypoxic and hypercapnic conditions.** (A-G) Under room-air conditions R26*Foxf1; Tie2*-cre neonates (*n*=16) show increased respiratory rate (A, *P*=0.01), reduced tidal volume (B, *P*=0.01) and similar minute ventilation levels (C) as compared to sibling controls (*n*=16). R26*Foxf1; Tie2*-cre animals show a reduced number of apneas (D, *P*=0.03) while average apnea length is similar (E). Significant reductions in instability are also seen in both interbreath interval (IBI) and tidal volume (F,G, *P*=0.006 and 0=0.0004). (H-N) Under hypoxic conditions (10% O_2_/90% N_2_) conditions, R26*Foxf1; Tie2*-cre neonates (*n*=11) show a reduced relative increase in respiratory rate compared to baseline room air respiratory rate (H, *P*=0.03), similar relative tidal volume increase (I), and a trend towards a reduced relative increase in overall minute ventilation (J, *P*=0.07) as compared to sibling controls (*n*=10). R26*Foxf1; Tie2*-cre neonates show similar numbers of apneas (K), and matched average apnea length (L) and levels of instability in both inter-breath interval (IBI) and tidal volume (M,N). (O-U) Under hypercapnia (5% CO_2_/21% O_2_/74% N_2_), R26*Foxf1; Tie2*-cre neonates (*n*=9) show a reduced relative increase in respiratory rate (O, *P*=0.04), matched increase in tidal volume (P), and a statistically insignificant reduced relative increase in minute ventilation (Q) as compared to sibling controls (*n*=10). R26*Foxf1; Tie2*-cre pups also show matched number (R) and length of apneas (S) and levels of instability in both IBI and volume (T,U). Data are shown as mean±s.e.m.

**Fig. 5.**
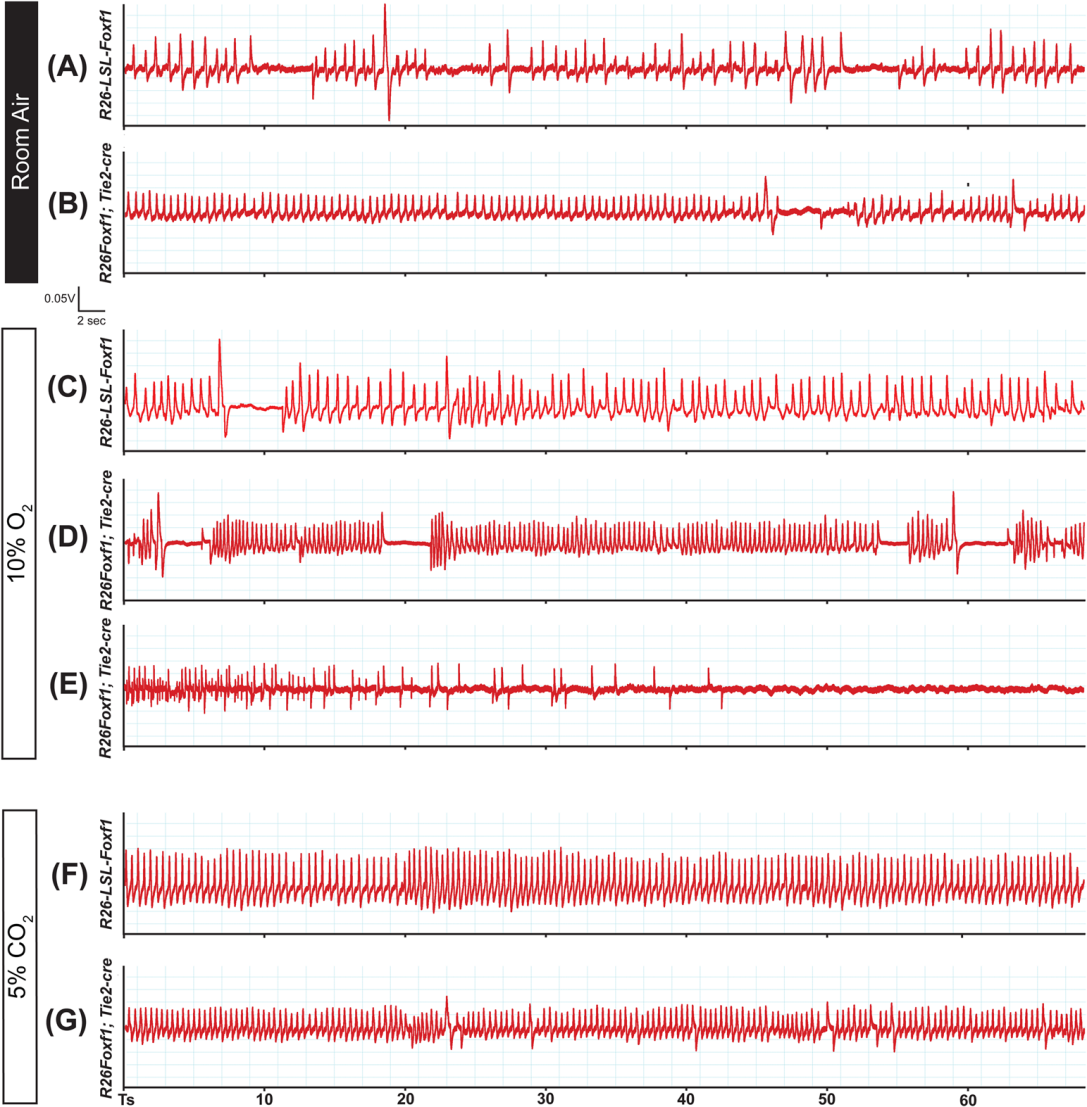
**Representative traces from R26*Foxf1; Tie2-*cre P0.5 neonates and sibling controls under room air, hypoxic, and hypercapnic conditions.** (A,B) Under room air conditions, sibling controls (A) show variability in both interbreath interval (IBI) and tidal volume with high numbers of apneas. In contrast, R26*Foxf1; Tie2-*cre neonates (B) show increased respiratory rate, reduced tidal volume, and more regular breathing with fewer apneas. (C-E) Under hypoxic conditions, apart from changes in amplitude, sibling controls (C) and R26*Foxf1; Tie2-*cre neonates (D) have similar respiratory traces, however, three out of eleven R26*Foxf1; Tie2*-cre animals ceased breathing (see E for example) and resumed once they were returned to room air conditions. (F,G) Under hypercapnic conditions, apart from differences in amplitude, R26*Foxf1; Tie2*-cre neonates and their sibling controls show similar patterning in breathing.

Respiratory function in P0.5 pups was tested under room air, hypoxic, and hypercapnic conditions using head-out plethysmography (Fig. 4). Under room-air conditions, the R26*Foxf1; Tie2-*cre pups showed significantly higher respiratory rates (V_f_) (Fig. 4A) while tidal volume per breath (V_T_) was significantly lower than in sibling controls (Fig. 4B). Total respiratory output per minute (V_E_) matched that of sibling controls (Fig. 4C). Examination of R26*Foxf1; Tie2-*cre respiratory traces showed a significant decrease in the number of apneas, but the average length of apneas was not significantly different (Fig. 4D,E). Respiratory traces also showed that R26*Foxf1; Tie2-*cre animals had more regular breathing patterns, as they had significant reductions in measures of variability in interbreath interval (IBI) and breath volume (Fig. 4G,H, see Fig. 5A,B for representative respiratory traces, and Fig. S8A,B for representative Poincaré plots).

When challenged with hypoxia, R26*Foxf1; Tie2-*cre animals showed a significant decrease in the relative change in respiratory rate as compared to sibling controls (Fig. 4H). As overall tidal volume did not significantly change as compared to room air values (Fig. 4I), R26*Foxf1; Tie2-*cre animals showed a trend of reduced relative increase in overall minute ventilation as well as reduced absolute V_T_ and V_E_ (Fig. 4J, Fig. S9B,C). R26*Foxf1; Tie2*-cre animals also showed a trend toward an increase in apneas but it was not significant (Fig. 4K). There was no difference in IBI or volume variability (Fig. 4M,N; see Fig. 5C,D for representative traces and Fig. S8C,D for representative Poincaré plots). Interestingly, while most R26*Foxf1; Tie2*-cre animals maintained a steady breathing pattern upon hypoxic exposure, three out of the 11 animals ceased breathing (see Fig. 5E for example trace) but resumed upon re-exposure to room air.

Upon a mild 5% hypercapnic challenge, R26*Foxf1; Tie2-*cre animals showed a blunted response with a reduction in the relative increase in V_f_ as compared to sibling controls (Fig. 4O), but no difference in relative change of V_T_ (Fig. 4P) or V_E_ (Fig. 4Q), resulting in a trend of reduced absolute V_T_ and V_E_ (Fig. S9E,F). Under the increased drive mediated by hypercapnia, there were no significant differences in the number or length of apneas (Fig. 4R,S) and no significant differences were found in IBI and volume variability (Fig. 4T,U).

### E18.5 R26*Foxf1; Tie2*-cre lung gene expression analysis

**Fig. 6.**
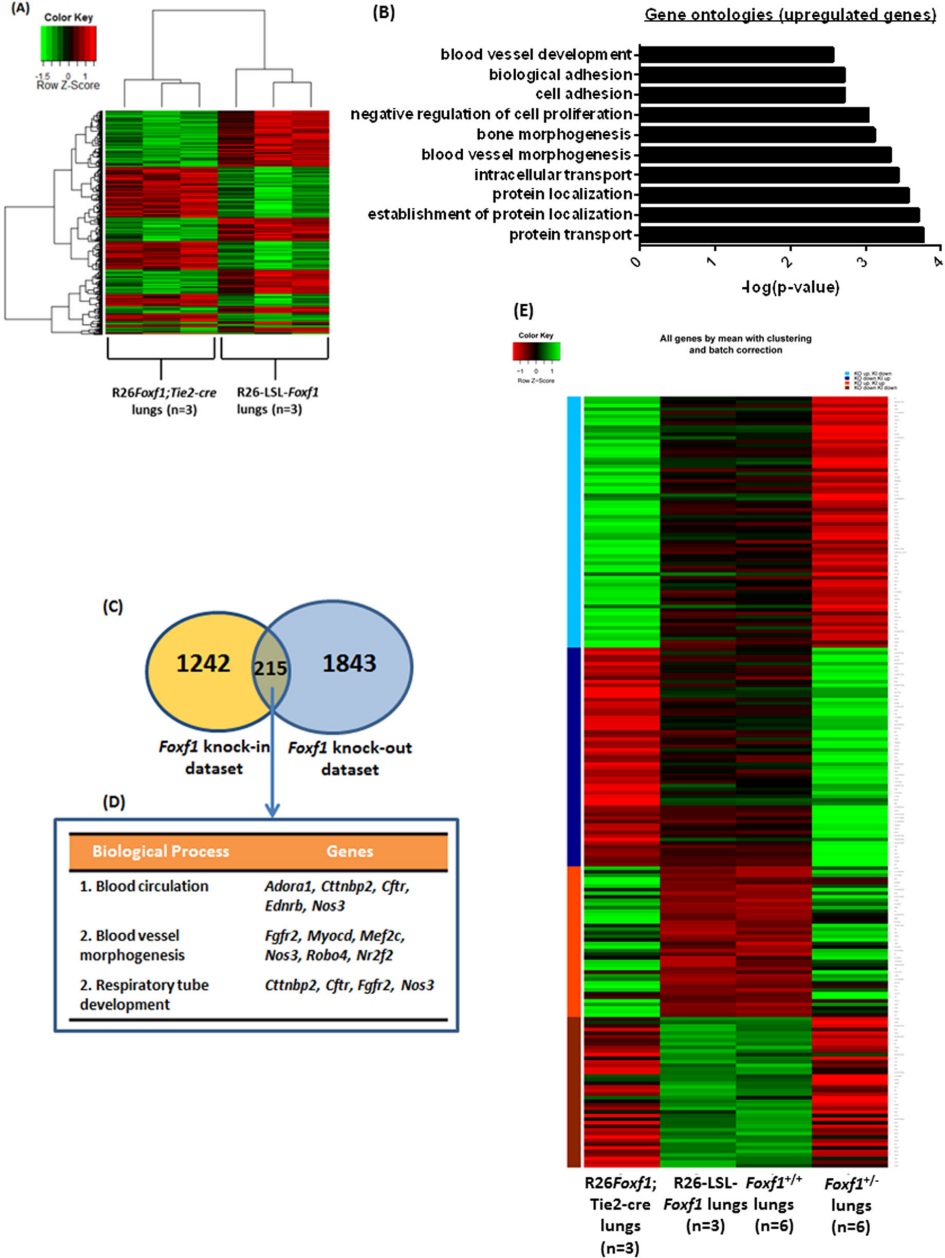
**E18.5 R26*Foxf1; Tie2-*cre lung microarray analysis: heat map, clustering, GO and pathway analyses.** (A) Heat map for genes differentially expressed (FDR <0.05, fold change ≥1.2 or ≤−1.2) between R26*Foxf1; Tie2-*cre lungs (*n*=3) and littermate control lungs (*n*=3). Decreased gene expression is shown in green and increased gene expression is shown in red. R26*Foxf1; Tie2-*cre lung samples and control lung samples cluster as two separate groups. (B) Top significant gene ontology (biological processes) terms associated with deregulated genes. Biological processes with the highest –log (*P*-values) are associated with highest enrichment. (C) Comparison of *Foxf1* knock-in and knock-out lung microarray datasets identified 215 overlapping deregulated genes. (D) List of commonly deregulated genes associated with biological processes of interest including blood circulation, blood vessel development, and lung development. (E) Heat map showing gene expression trends between the commonly regulated genes in the two datasets. The light blue bar represents genes upregulated in the *Foxf1* knock-out dataset and downregulated in the *Foxf1* knock-in dataset; dark blue bar represents genes down regulated in the knock-out dataset and upregulated in the knock-in dataset; orange bar represents genes upregulated in both datasets, and the red bar represents genes downregulated in both the datasets.

Statistical analyses of transcriptomes from microarray studies showed 1242 deregulated genes in R26*Foxf1; Tie2-*cre lungs compared to lungs from littermate controls [false discovery rate (FDR) <0.05, fold-change ≥1.2 and ≤−1.2] (Fig. 6A). 519 genes (41.79%) were down-regulated and 723 genes (58.21%) were up-regulated (Table S1). Database for Annotation, Visualization, and Integrated Discovery (DAVID) (Huang et al., 2009) analyses identified gene ontology (GO) terms related to protein transport, protein localization, cell adhesion, and blood vessel morphogenesis to be associated with the deregulated genes (Fig. 6B). The microarray data was verified for the genes *Igfbp3*, *Pparg*, and *Rcan1* using qRT-PCR (Fig. S10). Using a threshold of 1.2-fold-change, comparison of the R26*Foxf1*;*Tie2*-cre microarray dataset with our previously published *Foxf1* knock-out P0.5 lung dataset (Sen et al., 2014) revealed 215 genes to be commonly deregulated in both datasets (*P*-value for overlap: 6.5×10^−13^; Fig. 6C). Blood circulation, blood vessel development, and lung development were biological processes of interest found to be associated with the commonly deregulated genes in both the datasets (Fig. 6D). 165 out of 215 genes exhibited reciprocal gene expression trends, indicating that they could be direct targets of FOXF1 (*P*-value for reciprocal gene trends: 7.6×10^−16^; Fig. 6E; Table S3).

### E18.5 FOXF1 ChIP-seq analysis reveals targets involved in vasculature development

**Fig. 7.**
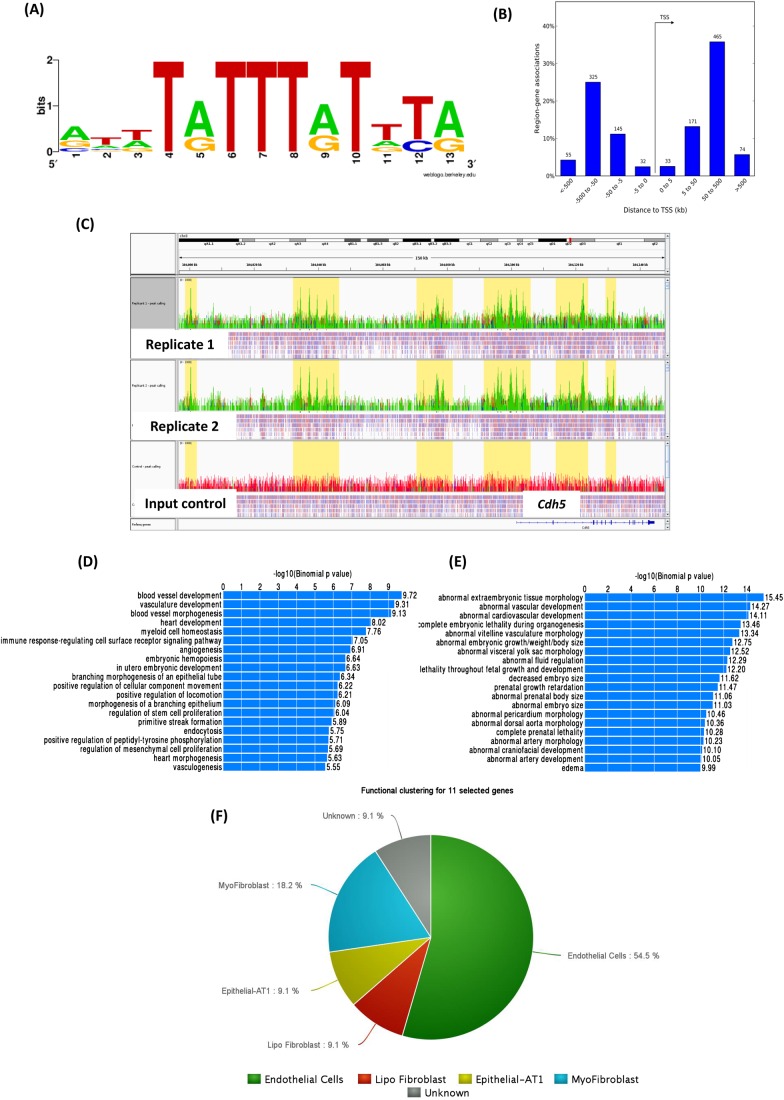
**E18.5 lung ChIP-seq analysis.** (A) Consensus FOXF1 binding motif identified from analysis of DNA sequences underlying ChIP-seq peaks. (B) Distribution of FOXF1 binding sites with respect to TSS, and percentage of region-gene associations. (C) Representative peaks for binding sites at the gene *Cdh5* in two biological replicates compared to input control. (D) Top biological processes enriched for genes associated with FOXF1 binding sites. (E) Top mouse phenotypes enriched for genes associated with FOXF1 binding sites. (F) Pie-chart showing functional classification from LungGENS common to FOXF1 ChIP-seq, *Foxf1* knock-in and *Foxf1* knock-out datasets.

Using a threshold of 5×10^−7^ for irreproducible discovery rate (IDR), ChIP-seq analysis identified 697 significant peaks for FOXF1 binding sites in E18.5 wild-type lungs. 1033 genes were found to be associated with these peaks (Table S4). Distribution of the peaks with respect to transcription start sites (TSS) is shown in Fig. 7A, with most peaks located 50 to 500 kb upstream or downstream to the TSS. Representative peaks in both biological replicates compared to the input control near the gene *Cdh5* are shown in Fig. 7B. Functional analysis using the Genomic Regions Enrichment of Annotations Tool (GREAT) (McLean et al., 2010) revealed enrichment of biological processes related to vasculature development, heart development, and embryonic development (Fig. 7C). Similar analysis for abnormal mouse phenotypes showed enrichment of phenotypes such as abnormal vascular, cardiovascular and embryonic development (Fig. 7D). Overlaying the FOXF1 ChIP-seq data with the R26*Foxf1*;*Tie2*-cre and *Foxf1*^+/*−*^ lung microarray datasets identified 11 common genes: *Arhgap18*, *Sox11*, *Zswim6*, *Tnfrsf19*, *Ednrb*, *Ghr*, *2510009E07Rik*, *Ostf1*, *Smarca2*, *Slit2* and *Nup210* (Fig. 7E). This layered and multi-step analysis further narrows down the list of potential direct targets of FOXF1 in the developing lung.

## DISCUSSION

In contrast to *Foxf1* haploinsufficiency, the phenotypic effects of the increased dosage of *Foxf1* during murine embryonic development are largely unknown. Previous studies include *Foxf1*-enforced expression *in vitro*, shown to repress hematopoiesis (Fleury et al., 2015), and overexpression of *Foxf1* in murine skin under the control of the basal cell-specific promotor Keratin 5 (*Krt5*), resulting in severe hair loss, increase in size and number of sebaceous glands, and growth failure of these animals ([Bibr BIO019208C54][Bibr BIO019208C55]). The R26-LSL-*Foxf1* line described here is a novel mouse model to overexpress *Foxf1* in a time- and tissue-specific manner.

*Tie2*-cre-mediated overexpression of *Foxf1* beginning at E7.5 in endothelial and hematopoietic lineages resulted in a combination of pulmonary, vascular, lymphatic and platelet defects. At E15.5, 35% of the heterozygous R26*Foxf1*; *Tie2*-cre embryos showed localized subcutaneous hemorrhages and edema, indicating a vascular fragility defect. Moreover, whole-body overexpression of *Foxf1* using *CMV*-cre also led to embryonic lethality, with R26*Foxf1*; *CMV*-cre embryos exhibiting hemorrhages. We elected to specifically focus on the effects of *Foxf1* overexpression in the lungs because of the pulmonary phenotypes seen in patients with ACDMPV. The majority of R26*Foxf1*; *Tie2*-cre pups died within 24 h of birth, due to respiratory failure.

The lungs of the heterozygous R26*Foxf1*; *Tie2*-cre pups were smaller compared to those of their littermate controls. qRT-PCR showed a 1.7-fold overexpression of *Foxf1* in E18.5 R26*Foxf1*; *Tie2*-cre lungs and an increase in the amount of the FOXF1 protein was also observed by IHC in E18.5 lungs. The lungs were found to be immature by histopathological evaluation and were associated with decreased expression of the vascular marker FLK1, but not the epithelial marker proSPC. In mice, a wet LW:BW ratio less than 4% defines pulmonary hypoplasia (Seegmiller et al., 1986), indicating that the R26*Foxf1*; *Tie2*-cre lungs with a wet LW:BW ratio of approximately 3% are hypoplastic. In support of this notion, micro-CT imaging showed that the E18.5 average lung volume and lung surface to body weight ratios of R26*Foxf1*; *Tie2*-cre embryos were approximately half and one-third smaller, respectively, compared to control lungs.

Examination of respiratory function revealed multiple deficits under baseline (room air), hypoxic and hypercapnic conditions. Under baseline conditions, P0.5 R26*Foxf1*; *Tie2-*cre mice had a reduced V_T_, likely due in part to the reduced volume of the hypoplastic lungs. However, an increase in V_f_ was compensatory and resulted in equivalent minute ventilation V_E_. The increase in respiratory drive is also the most probable reason behind the reduced number of apneas and reduced periodic and volume instability in comparison to the typical irregular breathing pattern of sibling controls (Hilaire and Duron, 1999), with no additional change to apnea length. Despite the equivalent ventilatory output, R26*Foxf1*; *Tie2*-cre pups still exhibit lethality, suggesting that changes in respiratory rate are insufficient to overcome the observed pulmonary and vascular defects, an observation supported by our hypoxic studies. Whereas the *Tie2-*cre is expressed in the neurovasculature, central- or peripheral-neural- or glia-specific expression has not been reported or observed in our hands. We hypothesize that increasing pulmonary-vascular dysfunction results in failure of adaptive regulation through greater levels of oxygen desaturation that ultimately leads to cardiopulmonary failure.

Microarray analyses of the E18.5 R26*Foxf1*; *Tie2*-cre lung transcriptome showed enrichment of genes related to protein transport, protein localization, blood vessel development, and cell adhesion. Some of the downregulated genes included *Igfbp3*, *Pparg*, *Rcan1* and *Prkcdbp*. *IGFBP3* and *PRKCDBP* are also inversely upregulated in ACDMPV lungs (Sen et al., 2014), suggesting that these genes might be relevant to the role of *FOXF1* in the pathology of ACDMPV. Downregulation of *Igfbp3* is associated with nitrofen-induced pulmonary hypoplasia (Ruttenstock et al., 2010). Interestingly, *Pparg* agonists enhance lung maturation and attenuate hypoxia-induced inhibition of lung development (Wang et al., 2009; Nicola et al., 2011), whereas *Rcan1* is implicated to play a role in angiogenesis as a regulator of calcineurin (Riper et al., 2008), and is activated by *Vegf* (Holmes et al., 2010). Some of the upregulated genes included *Fgfr2*, *Egfl7* and *Robo4*. *Fgfr2* upregulation has been described to be associated with nitrofen-induced pulmonary hypoplasia (Friedmacher et al., 2012). *Egfl7* overexpression in mice has been reported to cause embryonic lethality due to impaired angiogenesis (Nichol et al., 2010), whereas *Robo4* is a vascular specific receptor known to inhibit endothelial migration (Park et al., 2003). Furthermore, comparison of the genes deregulated in the R26*Foxf1*; *Tie2*-cre lung dataset with the genes differentially expressed in the dataset associated with the heterozygous loss of *Foxf1* in P0.5 lungs (Sen et al., 2014) showed involvement of genes associated with a variety of biological processes, including blood circulation, vasculature development and lung development. Of note, 165 genes showed reciprocal trends in gene expression, when compared between the two datasets. These genes are potential targets of FOXF1 in the lung, as their expression changes reciprocally with the loss or gain of *Foxf1*.

ChIP-seq analysis in E18.5 wild-type lungs identified binding of FOXF1 in the proximity of genes involved in biological processes such as blood vessel, cardiovascular and embryonic development. Among the genes associated with multiple FOXF1 binding sites, *Nrp1* signaling has previously been shown to be essential for fetal pulmonary development (Joza et al., 2013). Interestingly, mice homozygous for a knock-out allele of *Sdpr* (*Cav2*) exhibit abnormal caveolae formation in the lung endothelium affecting endothelial cell function (Hansen et al., 2013). Similarly *Cdh5* and *Itgb1* are genes involved in endothelial cell development. Recently, it was shown that *Itgb1* controls *Cdh5* localization and blood vessel stability (Yamamoto et al., 2015). Another forkhead gene, *Foxa2*, has been described to be required for the transition to breathing at birth (Wan et al., 2004). When the FOXF1 ChIP-seq data were compared to the *Foxf1* knock-in and knock-out microarray datasets, 11 genes were found to have binding sites for FOXF1 and were reciprocally deregulated in the microarray datasets. These included the genes *Arhgap18*, *Sox11*, *Zswim6*, *Tnfrsf19*, *Ednrb*, *Ghr*, *2510009E07Rik*, *Ostf1*, *Smarca2*, *Slit2* and *Nup210*. Interestingly, *Sox11*^−/−^ mice exhibit lung hypoplasia and die at birth (Sock et al., 2004). *Ghr* signaling is involved in early lung growth, oxidative protection, and lipid metabolism in the developing lung (Beyea et al., 2006). These genes could potentially be direct targets of FOXF1 in the embryonic and early postnatal lung.

In conclusion, the R26*-*LSL-*Foxf1* mice develop lung and vascular defects when crossed to the *Tie2*-cre line that activates recombination in endothelial and hematopoietic lineages. The defects manifest as clear functional deficiencies in neonate respiratory function. Our study shows that *Foxf1* is highly dosage sensitive, with both loss- and gain-of-function of *Foxf1* having implications in development and disease conditions. Additionally, the dosage sensitivity of *Foxf1* suggests that conventional gene therapy approaches to treat ACDMPV and other *FOXF1* related disorders may not be successful. Instead, alternative approaches that manipulate targets downstream of FOXF1 would need to be investigated.

## MATERIALS AND METHODS

### Animal care

All mouse experiments were carried out under the approval of the Institutional Animal Care and Use Committee (IACUC) at Baylor College of Medicine (BCM). Mice were housed in the Transgenic Mouse Facility (barrier level 3) under the care of the Center for Comparative Medicine (CCM), which is accredited by the Association for Assessment and Accreditation of Laboratory Animal Care International (AAALAC).

### Generation of the ROSA26 targeting construct

The ROSA26 *locus* is ubiquitously expressed; disruption of the endogenous transcript has no apparent phenotype (Zambrowicz et al., 1997) and is widely used as a reporter locus to determine expression from tissue-specific cre drivers (Srinivas et al., 2001) and to model targeted overexpression of oncogenes (Carofino et al., 2013).

*Foxf1* cDNA was amplified using Platinum Pfx polymerase (Thermo Fisher Scientific, Waltham, MA, USA) and the following primers: forward 5′-ACTAATTTAAAACCATGGACCCC-3′ and reverse 5′-ATTAGGTCGACTCACATCACACAC-3′. The resulting product was digested with *Dra*I and *Sal*I and ligated into the pENTR1A dual selection vector (Thermo Fisher Scientific, Waltham, MA, USA). Gateway recombination using LR Clonase II mix (Thermo Fisher Scientific, Waltham, MA, USA) was used to transfer the *Foxf1* fragment from the pENTR1A vector to pROSA26-DEST (Addgene plasmid 21189). Plasmid DNA was linearized with *BbvC*I.

### Chimeric mouse generation and breeding

Targeting vector DNA was electroporated into the C57BL/6N embryonic stem cell (ESC) line JM8A3 (Pettitt et al., 2009) by the Mouse Embryonic Stem Cell Core at BCM. Correctly targeted clones were microinjected into C57BL/6-*Tyr^c-Brd^* (albino) blastocysts and transplanted into pseudopregnant foster mothers by the Genetically Engineered Mouse (GEM) Core at BCM. Chimeric male offspring were crossed to C57BL/6-*Tyr^c-Brd^* females to test for germline transmission of the targeted ROSA26 allele, referred to here as R26-LSL-*Foxf1*. R26-LSL-*Foxf1* animals were crossed to the B6.Cg-Tg(*Tek*-cre)1Ywa/J (*Tie2*-cre) line (Kisanuki et al., 2001), obtained from the laboratory of Dr. Daryl Scott (BCM) and the B6.C-Tg(*CMV*-cre)1Cgn/J line (Schwenk et al., 1995). The *Tie2*-cre line is expressed in endothelial and hematopoietic cell lineages beginning at E7.5 and the X-linked *CMV*-cre line is likely activated before implantation during early embryogenesis and is expressed in all tissues, including germ cells.

### Southern blotting

Southern blotting was performed using P^32^ labeled probes as previously described (Justice et al., 1994). Probes on both the 5′ and 3′ side of the insert were utilized to test the accuracy of ROSA26 targeting. Probes were amplified from mouse genomic DNA via PCR with AmpliTaq Gold 360 (Thermo Fisher Scientific, Waltham, MA, USA) using primers 5′-CGCCTAAAGAAGAGGCTGTG-3′ / 5′-ACTCAACTTGCACGAACACG-3′ (5′ probe) and 5′-ACAGAGCATTGGCATTTTCC -3′ / 5′-AGCCAGTCCAAGAGAAAGCA -3′ (3′ probe). Genomic DNA from ESC clones was digested with *Eco*RV (WT fragment 11.5 kb, targeted fragment 4 kb) for the 5′ blot and *Pvu*II (WT fragment 5.9 kb, targeted fragment 2.2 kb) for the 3′ blot.

### Genotyping PCR

Genomic DNA was prepared from tail biopsies for genotyping. Animals were genotyped using primers specific for the Cre transgene: 5′-GCCAGCTAAACATGCTTCATC-3′/5′-ATTGCCCCTGTTTCACTATCC-3′, full-length R26-LSL-*Foxf1* 5′-TTCCCTCGTGATCTGCAACT-3′/5′-GCCAGAGGCCACTTGTGTAG-3′, Cre-deleted R26*Foxf1:* full-length R26PR-F/5′-AGGTAGTTCGCCTTGTCCTG-3′-R, and the WT ROSA26 locus: full-length R26PR-F/5′-CCGACAAAACCGAAAATCTG-3′-R. PCR was performed using *Taq* DNA Polymerase (Thermo Fisher Scientific, Waltham, MA, USA).

### Endothelial cell flow sorting

E18.5 lungs (R26*Foxf1*; *Tie2*-cre and control +; *Tie2*-cre) were dissected in 3 cm dishes and minced finely with forceps and scissors. Then 2 ml of the digestion medium (HBSS supplemented with 0.05% Trypsin, 0.1% Collagenase and 25 µM HEPES) was added to the dish and the tissue was incubated for 1 h at 37°C. The reaction was stopped by addition of cold MEM supplemented with 10% FBS. The tissue was then disaggregated by pressing through a 40 µm nylon mesh. Cells were centrifuged at 290 ***g*** (1200 rpm) for 10 min and the pellet was re-suspended in 250 µl PBS supplemented with 1 mM EDTA, 25 mM HEPES, and 1% FBS to ∼10^6^ cells/ml. Endothelial cells were stained with anti-CD31 (FITC conjugated) antibodies (Abcam, Cambridge, MA, USA) at a 1:50 dilution for 1 h at 4°C in the dark. Washing was done three times in the same buffer that was used for staining. Sorting of CD31-positive cells was done on FACSAriaII cell sorter (BD Biosciences, San Jose, CA, USA) using a 70 µm nozzle. Cells were collected directly into QIAzol and processed for RNA isolation using miRNeasy Mini Kit (Qiagen, Hilden, Germany).

### Immunohistochemistry

Formalin fixed E18.5 lungs were paraffin embedded and sectioned according to standard procedures. Paraffin sections were stained using antibodies specific to FOXF1 (Malin et al., 2007), FLK1 (Santa Cruz Biotechnology, Dallas, TX, USA), proSPC (Ustiyan et al., 2012) and α-SMA (Abcam) as described (Ustiyan et al., 2009; Wang et al., 2010).

### Hematology measurements

For hematology measurements trunk blood was obtained from P1.5 pups by decapitation. CBCs were performed by the Comparative Pathology Laboratory at BCM on an Advia 120 automated hematology system (Siemens) on samples pooled from 2-3 pups.

### Micro-CT imaging

E15.5 and E18.5 embryos were imaged by micro-CT at the Optical Imaging and Vital microscopy (OIVM) core at BCM. Embryos were prepared by a method called STABILITY as described previously (Wong et al., 2013). In short, embryos were fixed in 4% PFA; hydrogel stabilized using acrylamide and stained with 0.1N iodine. 3D datasets for each embryo were acquired using SKYSCAN 1272 micro-CT scanner (Bruker). Images were obtained using a 0.5 mm aluminum filter with a rotation step of 0.2 at a resolution of 11 μm. The acquired datasets were reconstructed using the NRecon software (Bruker) and visualized using the CTVox software (Bruker).

### Lung volume quantification

Individual lung images were extracted using the region of interest (ROI) function of the CTAn software (Bruker) from reconstructed NRecon datasets to create volume of interest (VOI) datasets for each embryo. The VOI lung datasets were visualized using the Imaris software (BitPlane) and lung volumes calculated using the surface rendering function in Imaris.

### Neonatal head-out pneumotachography

P0.5 respiration was measured in a custom built head-out mask-pneumotachograph system as described previously (Cummings and Frappell, 2009), that was engineered and machined for a minimum of dead space to increase sensitivity. Additional facemask ports were engineered for gas flow-through and calibration. For calibration and experiments, room air or mixed gasses were drawn through the mask-pneumotachograph by a vacuum pump attached to the gas flow-through port. Mixed gasses were supplied by a magnetically coupled bell housing that rested over the end of the pneumotachograph while remaining partially open to the room to avoid pressure fluctuations from the inflowing gas. All measurements were done between 7 am and 3 pm on the day of birth.

Prior to an experiment, the facemask was sealed with a piece of nitrile rubber. Ventilation was calibrated as a series of 20 µl pipetman injections into an empty facemask at a rate of 3 Hz. The rate of gas flow-through was continuously controlled via two rotameters in series.

For experimental assays, a small opening was made in the nitrile rubber to fit the snout (nose and mouth) of a P0.5 mouse. The mouse was affixed to the facemask with Impregum F, Polyether Impression material (Patterson Dental, St. Paul, MN, USA). The mouse rested on a platform attached to the facemask that fit inside a temperature controlled chamber to maintain the mouse pup at 36°C.

Pneumotachograph pressure changes and chamber temperature were recorded with LabChartPro (AD Instruments, Colorado Springs, CO, USA) in real time. The pneumotachograph trace was integrated to produce a respiratory waveform. Waveforms were analyzed offline to determine respiratory rate (V_f_), tidal volume (V_T_), minute ventilation (V_E_) and pattern analysis.

After attachment to the face mask, mice were allowed to acclimate for 10 min in room air. Data was recorded for another 20 min under room air conditions and then switched for an additional 20 min to either a mix of 10% O_2_/90% N_2_ for hypoxia or a mix of 5% CO_2_/21% O_2_/74% N_2_ for hypercapnia before returning to room air for 20 min.

Respiratory waveforms were collected when the neonate was at rest and readings were free from movement artifacts. A minimum of 1 min cumulative data compiled from traces at least 10 s long from the last 5 min of a given experimental condition were analyzed. Apart from integration, no filtering, smoothing or other manipulations were applied to the pressure waveform. Tidal volume (V_T_) was determined by comparing peak (mV) height to calibration injections (mV/µl).

Poincaré plots and apnea measurements were determined using 10 min of movement-free traces from each breathing condition. Apneas were defined as an interbreath interval (IBI) that was longer than 1.5 s. The coefficient of variation (CV) of the IBI and amplitude was also calculated from the same 10-minute trace compilation of each breathing condition (standard error IBI or amplitude/mean IBI or amplitude).

### Gene expression arrays

Illumina mouse WG-6 v2.0 expression Beadchip analysis was performed on three R26*Foxf1;Tie2-*cre E18.5 lung RNA samples and three control R26-LSL-*Foxf1* E18.5 lung RNA samples. 500 ng of total RNA was labeled using Illumina TotalPrep RNA Amplification Kit (Thermo Fisher Scientific, Waltham, MA, USA) and hybridized as per manufacturer's instructions. The array data were analyzed using the lumi bioconductor package (Du et al., 2008), normalized by robust spline normalization and transformed using variance stabilization transformation (VST) as previously described (Sen et al., 2014). A two-sample *t*-test was applied to determine differentially expressed genes between R26*Foxf1; Tie2-*cre and the R26-LSL-*Foxf1* lung groups. Differential expression *P*-values were adjusted for false discovery rates (FDR). Fold changes were calculated using reverse VST. Although our sample size was relatively small for parametric tests, we justified the use of *t*-tests in this study by large effect sizes of our analysis (DeWinter, 2013). Database for Annotation, Visualization, and Integrated Discovery (DAVID) (Huang et al., 2009) was used for gene ontology and pathway analyses.

### Quantitative RT-PCR

Total lung RNA was reverse transcribed to cDNA using the SuperScript III First-Strand Synthesis System (Thermo Fisher Scientific, Waltham, MA, USA) and amplified using the Power SYBR Green PCR Master Mix (Thermo Fisher Scientific, Waltham, MA, USA). Primers used for qRT-PCR validation of the mouse microarray are listed in Table S2. Amplification and data analysis were conducted on an ABI 7900HT Fast real time PCR System (Thermo Fisher Scientific, Waltham, MA, USA). Relative gene expression was calculated using the ΔΔC_T_ method (Pfaffl, 2001). To determine *Foxf1* overexpression, TaqMan probes (Thermo Fisher Scientific, Waltham, MA, USA) were used for *Foxf1* and *Gapdh* (internal control). Amplification was performed using the TaqMan Universal PCR Master Mix (Thermo Fisher Scientific, Waltham, MA, USA).

### ChIP-seq assay and analysis

Two biological replicates of pooled E18.5 lungs (*n*=3) were crosslinked with 37% formaldehyde to a final concentration of 1%. ChIP was performed using the SimpleChIP Enzymatic Chromatin IP Kit (Cell Signaling, 9005S). Micrococcal nuclease-digested chromatin was further sonicated using Diagenode Bioruptor with 20 pulses of 15 s on and 15 s off at high power to yield sheared chromatin. Ten micrograms of chromatin was used per immunoprecipitation with 7 µg of FOXF1 antibody (Ren et al., 2014) (AF4798: R&D Biosystems, Lot#B1508). ChIP-grade normal rabbit IgG #2729 and Histone H3 (D2B12) XP^®^ Rabbit mAb (ChIP formulated) from Cell Signaling Technology were used as negative and positive controls, respectively; 2% input was used as a control. The Genomic and RNA Profiling (GARP) Core at BCM conducted sample quality checks using the NanoDrop spectrophotometer, Invitrogen Qubit 2.0 DNA quantitation assay and Agilent Bioanalyzer. The Rubicon ThruPlex DNA-Seq library preparation system was used to prepare ChIP-Seq libraries for sequencing on the Illumina HiSeq sequencing system. Sequence reads were mapped on to the mm10 genome using Bow-tie2 (Langmead and Salzberg 2012). Percentage of uniquely mapped reads were 77.85, 73.10 and 73.10%, respectively, for the two biological replicates and input control samples, acceptable according to Bailey et al. (2013). Peak calling procedure was performed using the Model-based Analysis of ChIP-Seq (MACS2) tool (Zhang et al., 2008). The assessment of peak calling was done using IDR-score (Li et al., 2011). The consensus FOXF1 binding motif was identified from the DNA sequences underlying the peaks, using the WebLogo tool (http://weblogo.berkeley.edu/logo.cgi). Functional analysis of selected peak regions was performed using the GREAT tool (McLean et al., 2010) and classification of genes common to the ChIP-seq and microarray datasets was done using LungGENS (Du et al., 2015).

### Data analysis

Graphical and statistical analyses were conducted using Prism (GraphPad, La Jolla, CA). A χ^2^ test was used for analysis of timed matings and an unpaired *t*-test was used for all other analyses. Standard error of the mean is shown on all figures. Statistical significance was considered at *P*≤0.05.
